# Preparation of Squalene Oil-Based Emulsion Adjuvants Employing a Self-Emulsifying Drug Delivery System and Assessment of *Mycoplasma hyopneumoniae*-Specific Antibody Titers in BALB/c Mice

**DOI:** 10.3390/pharmaceutics11120667

**Published:** 2019-12-10

**Authors:** Rakesh Bastola, Jo-Eun Seo, Taekwang Keum, Gyubin Noh, Jae Woong Choi, Jong Il Shin, Ju Hun Kim, Sangkil Lee

**Affiliations:** 1 College of Pharmacy, Keimyung University, 1095 Dalgubeol-daero, Dalseo-gu, Daegu 42601, Korea; bastola777rakesh@gmail.com (R.B.); joeun0405@hanmail.net (J.-E.S.); gtk02@hanmail.net (T.K.); rhgyubin@naver.com (G.N.); 79182@naver.com (J.W.C.); 2 Komipharm International Co., Ltd., 17 Gyeongje-ro, Siheung-si, Gyonggi-do 15094, Korea; shinjongil00@gmail.com (J.I.S.); khjh99@komipharm.com (J.H.K.)

**Keywords:** self-emulsifying drug delivery system, squalene oil, emulsion adjuvants, *Mycoplasma hyopneumoniae*-specific antibody titers

## Abstract

In this study, a self-emulsifying drug delivery system (SEDDS) was employed to prepare novel squalene oil-based emulsion adjuvants. Deionized water, 0.01% and 0.02% (*w*/*v*) carbomer solutions of C-971P NF and C-940 grades were used to prepare emulsions containing 3%, 5% and 10% of squalene oil. Altogether 15 candidate emulsions were prepared and used as adjuvants for the delivery of a combination vaccine containing a porcine circovirus type 2 (PCV2) antigen and inactivated *Mycoplasma hyopneumoniae* (J101 strain) antigen. Most of the emulsions showed droplet sizes in the submicron range and maintained zeta potential values between −40 mV to 0 mV for six months, indicating good physical stability as a vaccine adjuvant. Emulsion-based candidate adjuvants prepared with SEDDS technology stimulated IgG, IgG1 and IgG2a like a currently commercially available adjuvant, Montanide ISA^TM^ 201, and they were safe and their *Mycoplasma hyopneumoniae*-specific antibody titers were considered as comparable with that of Montanide ISA^TM^ 201.

## 1. Introduction

In general, protein-based subunit vaccines are co-formulated with adjuvants to improve the immune responses of subunit antigens [1,2]. However, adjuvants are also used with inactivated pathogens to boost immunity [3,4,5]. Adjuvants reduce the dose of the antigen and required number of vaccine doses. Furthermore, they induce rapid and long-lasting immune responses [1]. Various adjuvants have been investigated for their adjuvant properties, e.g., mineral salts, oil-based emulsions, liposomes, virosomes, niosomes, saponins, immune-stimulating complexes, polymeric particles, cytokines, virus-like particles and bacterial derivatives, but only few of them are used in human and animal vaccines [6]. Emulsions are widely reported as vaccine delivery systems [7]. An emulsion (or microemulsion) is a mixture of immiscible liquids stabilized by surfactants [8,9,10]. Emulsions are classified into oil-in-water (o/w), water-in-oil (w/o) and multiple emulsions, namely, water-in-oil-in-water and oil-in-water-in-oil [11]. The quality of the immunogenic responses stimulated by o/w emulsions is substantially higher when compared to aluminum salt-based adjuvants; therefore, o/w emulsions have gained more attention as an adjuvant [12]. MF59 and AS03 are widely studied squalene oil based o/w emulsions that have been licensed for human use to deliver influenza vaccines [7]. High pressure homogenization like microfluidization and phase inversion are typical methods employed for manufacturing emulsions [13]. But here we focused to develop o/w emulsions using a simple self-emulsifying process that needs only simple stirring. Such a process is easy and economic. Self-emulsifying drug delivery system (SEDDS) is a lipid based drug delivery system that is suitable for delivery of drugs with poor aqueous solubility and low oral bioavailability. SEDDS is composed of oil, surfactants and co-surfactants in a suitable amount that can form an o/w emulsion upon exposure to the aqueous media [14]. Shah et al. demonstrated the preparation of emulsion adjuvants using a self-emulsifying process, which was accompanied with mixing, heating and filtering [15]. In this study, o/w emulsions were prepared with squalene oil, Span 80 and Cremophor ELP, using the SEDDS technique and used as an adjuvant with an antigen solution composed of a porcine circovirus (PCV) type 2 antigen and an inactivated *Mycoplasma hyopneumoniae* antigen. However, in this study, only *Mycoplasma hyopneumoniae*-specific antibody titers were determined. In addition, immunogenic responses of emulsion adjuvants prepared with different concentrations of carbomer solutions of grades C-971P NF and C-940 were also assessed.

## 2. Materials and Methods

### 2.1. Materials

Squalene oil was purchased from Alfa Aesar (Seoul, Korea), Span 80 from Tokyo Chemical Industry Co., Ltd. (Tokyo, Japan) and Cremophor ELP from BASF (Ludwigshafen, Germany). Two different grades of carbomers, C-971P NF and C-940, Montanide ISA^TM^ 201 and an antigen solution incorporating a PCV type 2 antigen and inactivated *Mycoplasma hyopneumoniae* (J101 strain, phosphatase (−), arginine hydrolysis (−), urease (−)) antigen were supplied from Komipharm Int’l Co., Ltd. (Siheung, Korea). DNA extraction kit and PCR premix were purchased from iNtron Biotechnology (Seongnam, Korea). Rabbit anti-Mouse IgG2a secondary antibody and horseradish peroxidase (HRP) was purchased from Thermofisher (Waltham, MA, USA), mouse IgG1 antibody (also known as goat anti-Mouse IgG1, HRP) from GeneTex (Hsinchu City, Taiwan) and goat anti-Mouse IgG (H+L) secondary antibody was purchased from Thermofisher (Waltham, MA, USA). The *Mycoplasma hyopneumoniae* antibody test kit was purchased from IDEXX (Westbrook, ME, USA). Other used reagents were of analytical grade.

### 2.2. Preparation and Characterization of PCV2 Antigen

*Spodoptera frugiperda* 9 (Sf9) cells are grown in suspension for 2 to 3 days and it was inoculated with PCV2 ORF2 expression baculovirus in the range of 1 to 10 MOI (multiplicity of infection) and incubated using a wave instrument at 27 °C constant temperature room. When the CPE (cytopathic effect) was 80% or more at 8 to 9 days of inoculation, the supernatant was harvested by centrifugation at 250× *g*, for 5 min to confirm the antigen titer. Thereafter, it was inactivated with formalin and stored at 4 °C until vaccine preparation. The prepared PCV2 antigen was observed by transmission microscopy (H-7600, Hitachi, Tokyo, Japan) and the molecular weight of the produced antigen was identified using SDS-PAGE.

### 2.3. Preparation and Characterization of Mycoplasma hyopneumoniae Antigen

#### 2.3.1. Culture Condition of *Mycoplasma hyopneumoniae* Antigen

The master seed in cryopreservation was primary cultured using *Mycoplasma hyopneumoniae* propagation medium and it was transferred to *Mycoplasma* broth, and finally incubated at 37 °C for ca. 7 days. Cultivation was terminated when the color of the medium changed to yellow, after which the incorporation of various bacteria was examined, and antigen titer was measured using the Color Change Unit (CCU) method. Briefly, after diluting the collected bacteria from 10^−1^ to 10^−9^ using sterile PBS, it was inoculated into each *Mycoplasma* broth and the concentration checked using the CCU method. After that, cells were harvested by inactivation with formalin and stored at 4 °C until vaccine preparation.

#### 2.3.2. Characterization Using Mycoplasma Selection Media

A total of 1 mL of culture material (*Mycoplasma hyopneumoniae*, J101 strain) was inoculated into 100 mL of *Mycoplasma* broth, followed by shaking and incubation at 37 °C for 14 days. After 14 days of inoculation, cells were transplanted to *Mycoplasma* agar, and incubated for 14 days at 37 °C and 4%–6% CO_2_ condition, and it was observed using a low magnification microscope (×100). *Mycoplasma gallisepticum* was used as a positive control group, and the concentration was 100 colony-forming units (CFU). The un-inoculated group was used as the negative control and boths were cultured under the same conditions.

#### 2.3.3. Identification of *Mycoplasma hyopneumoniae* Using Polymerase Chain Reaction (PCR)

The DNA of the positive control (*Mycoplasma hyopneumoniae*, KML Mh1 strain), the negative control (sterile distilled water) and the test (*Mycoplasma hyopneumoniae*, J101 strain) were extracted using a commercial DNA extraction kit (iNtron Biotechnology, Seongnam, Korea). A total of 1 μg of DNA and 1 μL of *Mycoplasma* p36 gene forward and reverse primer (10 pmol/μL, forward 5′-GGG CCG ATG AAA CCT ATT AAA ATA GCT-3′; reverse 5′-GCC GCG AAA TTA AAT ATT TTT AAT TGC ATC CTG-3′) were added to each PCR premix (iNtron Biotechnology, Seongnam, Korea) [16]. The PCR conditions used were as follow: One cycle of initial denaturation (for 15 min at 95 °C), 35 cycles of amplification (for 1 min at 94 °C, for 1 min at 50 °C and for 1.5 min at 72 °C) and one cycle of final extension (for 10 min at 72 °C). The PCR products (948 bp) were electrophoresed in 1.5% agarose gels and visualized with nucleic acid staining dye on the UV illuminator.

### 2.4. Construction of Pseudo-Ternary Phase Diagrams

Pseudo-ternary phase diagrams were constructed following the method described by Wang and Pal, with modifications [17]. Diagrams were prepared for different ratios of Span 80 to Cremophor ELP, including 1:4, 7:13 and 1:1. For each ratio, squalene oil and S_mix_ (mixture of Span 80 and Cremophor ELP) were mixed in ratios (*w*/*w*) from 1:9 to 9:1. To prepare emulsions and observe the transition of phases, deionized water was continuously added in small amounts to the mixture of oil and surfactants (SEDDS). Mixing was done in a beaker using a stirring bar and a magnetic stirring plate. All the experiments were conducted at room temperature. After each addition of water, categorization of phases was done on the basis of visual observation. Flowable dispersion was termed as emulsion (E) whereas non-flowable or slightly flowable masses were termed as gel (G). Categorization of phases and boundaries plotted in the diagrams depended upon the states of the dispersion observed after gentle stirring for few minutes, which can also be referred to as the non-equilibrium state.

### 2.5. Self-Emulsification Study

Evaluation of self-emulsification was done following the method described by Lee et al., with modifications [18]. In short, 1 mL of SEDDS formulation was added to 200 mL of deionized water at room temperature under gentle stirring. Then the self-emulsification property was determined by observing appearances of the final emulsion using a grading system where “A” refers to a clear or slightly bluish appearance, “B” refers to a bluish white appearance, “C” refers to a bright white appearance similar to milk, “D” refers to a dull, ash emulsion with slightly oily appearance and “E” refers to poor or minimal emulsification with large oil droplets present on the surface. Self-emulsification tests were also done using 0.01 and 0.02% (*w*/*v*) carbomer solutions of C-971P NF and C-940 grades.

### 2.6. Preparation of Emulsions

The ratio 7:13 of Span 80 to Cremophor ELP was chosen and emulsions containing 3%, 5% and 10% of squalene oil were prepared with deionized water. Required amounts of Span 80, Cremophor ELP and squalene oil were weighed and mixed in a beaker with the help of a stirring bar on the magnetic stirring plate. Then the calculated amount of deionized water was added in the beaker with continuous stirring to prepare the emulsion at room temperature. Likewise, emulsions were also prepared with 0.01% and 0.02% (*w*/*v*) carbomer solutions of two different grades namely, C-971P NF and C-940. Altogether 15 emulsions were prepared and their compositions are shown in Table 1. Emulsion adjuvants were preserved with formalin.

### 2.7. Particle Size and Zeta Potential of Emulsion-Based Adjuvants

Determination of mean particle size and zeta potential of emulsions were done using the dynamic light scattering (DLS) method using ZetaPALS (Brookhaven Instruments Corporation, New York, NY, USA) for a period of 6 months. All the emulsions were shaken well and properly diluted with deionized water before measurement.

### 2.8. Preparation of Vaccines

Emulsions were autoclaved and mixed in 1:1 ratio (*v*/*v*) with antigen solution containing a PCV type 2 antigen and an inactivated *Mycoplasma hyopneumoniae* antigen to prepare vaccines. In 2 mL of vaccine, the concentration of PCV type 2 antigen was above 100 units, inactivated *Mycoplasma hyopneumoniae* antigen was 1.0 × 10^8^ CCU and the concentration of the preservative was less than 0.2%.

### 2.9. ELISA Analysis Method for PCV2

The plate coated with PCV2 ORF2 antibody was removed at room temperature in advance and left for 1 h. After diluting the test sample and the standard sample 8000–64,000-fold with a dilution buffer, it was dispensed as 100 uL per well into the prepared plate, reacted at 37 °C for 1 h and washed 4 times using washing buffer. After a 100-fold dilution of the anti-PCV2 conjugate, the resultant was developed on a TMB (3,3′,5,5′-Tetramethylbenzidine) substrate and absorbance was measured at 450 nm. A standard curve was prepared using the standard sample, and the final quantitative value was calculated by substituting the optical density (OD) value of the sample.

### 2.10. Toxicity Study

A total of 120 BALB/c mice weighing 15–20 g were divided into 15 groups each containing 8 mice. Mice were injected intraperitoneally with 0.5 mL of emulsion adjuvants (SQ1–15) and observations were done for one week. This study was conducted following the approval of the IACUC (Institutional Animal Care and Use Committees: 2017-RD-0701, 4 July 2017) of Komipharm Int’l Co., Ltd., Republic of Korea.

### 2.11. Determination of Adjuvant-Dependent Antibody Subclasses

A total of 85 BALB/c mice weighing 15–20 g were divided into 17 groups each containing 5 mice. Mice in 15 different test vaccine groups were injected intraperitoneally with 0.5 mL of vaccines prepared from 15 emulsion adjuvants (SQ1–15). In the positive control group, each mouse was injected intraperitoneally with 0.5 mL of vaccine prepared from a commercial vaccine adjuvant—Montanide ISA^TM^ 201 (ISA)—and the remaining one group was used as the negative control (NC). All the mice were injected twice with the same dose of vaccine; the first vaccination was done at week 0 and the second after 2 weeks. Four weeks after the first vaccination, blood samples were collected from the intra-orbital vein of eye and tested for antibody titers. This study was also performed according to the approval of the IACUC (Institutional Animal Care and Use Committees: 2017-RD-0701, 4 July 2017) of Komipharm Int’l Co., Ltd., (Siheung, Korea).

*Mycoplasma hyopneumoniae*-specific IgG, IgG1 and IgG2a titers were detected by following an indirect ELISA method as described by Stipkovits et al., with modifications [19]. In short, a polystyrene ELISA plate (Corning Inc., New York, NY, USA) was coated with 0.5 μg/well of *Mycoplasma hyopneumoniae* antigens prepared in PBS (pH 7.4, 100 µL/well), covered with plate sealer and incubated at 37 °C for 30 min and then overnight in a moist chamber at 4 °C. After that, the plate was washed three times with ELISA buffer (PBS, pH 7.4, containing 0.05% Tween 20). Blocking buffer (PBS, pH 7.4, containing 1% BSA, 100 µL/well) was added, covered with plate sealer and incubated at 37 °C for 1 h. Then the plate was washed three times with ELISA buffer. A total of 100 µL of adequately diluted serum sample per well was added, covered with plate sealer and incubated at 37 °C for 1 h. Wells were aspirated and washed three times with ELISA buffer. 100 µL of adequately diluted secondary antibodies (goat anti-mouse IgG HRP or goat anti-mouse IgG1 HRP or rabbit anti-mouse IgG2a HRP) were added to each well, covered with plate sealer and incubated at 37 °C for 1 hr. Again, wells were aspirated and washed three times with ELISA buffer. A total of 100 µL of TMB substrate solution (SeraCare Life Sciences Inc., Milford, MA, USA) was added to each well and incubated at room temperature for 15 min under the protection of light. Finally, 50 µL of stop solution (2 N H_2_SO_4_) was added to each well and optical density (OD) was measured at 450 nm within 30 min, using a microplate reader (Molecular devices, VERSAmax microplate reader, South San Francisco, CA, USA).

### 2.12. Measurement of Mycoplasma hyopneumoniae-Specific Antibody Titers

To measure the level of *Mycoplasma hyopneumoniae* antibody, five mice weighing 15–20 g were used in each group. The vaccination group was inoculated subcutaneously with 1/10 dose (0.2 mL) for pigs. Two weeks later, mouse serum sample was collected from the eye using intra-orbital vein blood collection method and tested antibody titer using a commercial *Mycoplasma hyopneumoniae* antibody test kit (IDEXX, ME, USA). Briefly, *Mycoplasma hyopneumoniae* antigens pre-coated ELISA plate was brought to room temperature 30 min before use. Using the sample diluent, the control serum was diluted 10-folds and the experimental serum was prepared by diluting 512-folds. Standard negative serum was prepared without dilution. 100 µL of adequately diluted serum sample per well was added, covered with plate sealer and incubated at room temperature for 30 min. After that, aspirated each well and washed three times with washing solution. 100 µL of adequately diluted secondary antibodies (goat anti-mouse IgG HRP conjugate) were added to each well, covered with plate sealer and incubated at room temperature for 30 min. Again, wells were aspirated and washed three times with washing solutions. TMB substrate solution (SeraCare Life Sciences Inc., Milford, MA, USA) was added to each well and incubated at room temperature for 15 min under the protection of light. Finally, 50 µL of stop solution was added to each well and optical density (O.D) was measured at 650 nm within 30 min, using a microplate reader. The OD of the non-vaccinated control group should be lower than that of the standard negative serum, and the OD of the vaccinated group diluted 512 times should be higher than that of the standard negative serum.

### 2.13. Statistical Analysis

Data were analyzed using one-way analysis of variance (ANOVA) followed by a Dunnett’s test with the help of GraphPad Prism 5 software.

## 3. Results

### 3.1. Characterization of PCV2 Antigen Formation

**Figure 1:**
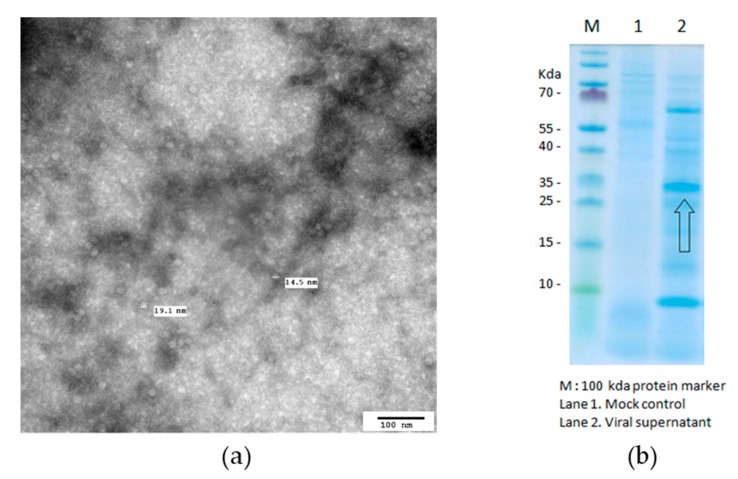
Transmission microscopic image of the PCV2 antigen (**a**) and SDS-PAGE result for PCV2 (**b**) where M is the 100 kDa protein marker, Lane 1 is the mock control and Lane 2 is the viral supernatant.

The electron micrograph of the PCV2 antigen revealed that PCV2 was prepared successfully as VLP (virus-like particle) antigen with a diameter of 14.5~19.1 nm (Figure 1a). From SDS-PAGE, it was found that the target band was observed at around 30 KDa (Figure 1b). PCV2 ORF2 protein antigen was quantified by ELISA, and the calibration curve showed good linearity (*Y* = 0.029*X* − 0.011, *R*^2^ = 0.999). Results are showing that PCV2 was formed successfully.

### 3.2. Characterization of Mycoplasma hyopneumoniae Antigen Formation

#### 3.2.1. Measurement of *Mycoplasma hyopneumoniae* Antigen Titer

The titer was confirmed using a color changing unit (CCU) method and the measured concentration was 10^9.5^ CCU/mL.

#### 3.2.2. Optical and Microscopic Observation for the Characterization of *Mycoplasma hyopneumoniae*

In the positive control group inoculated with *Mycoplasma gallisepticum*, the color of the medium changed to yellow after 14 days of inoculation into *Mycoplasma* broth. In case of *Mycoplasma hyopneumoniae*, there was a color change in the *Mycoplasma* broth like the positive control. Colony formation ability on agar was similar to that of the positive control, and proliferation was successful (Table 2).

#### 3.2.3. Identification of *Mycoplasma hyopneumoniae* Using PCR Method

**Figure 2:**
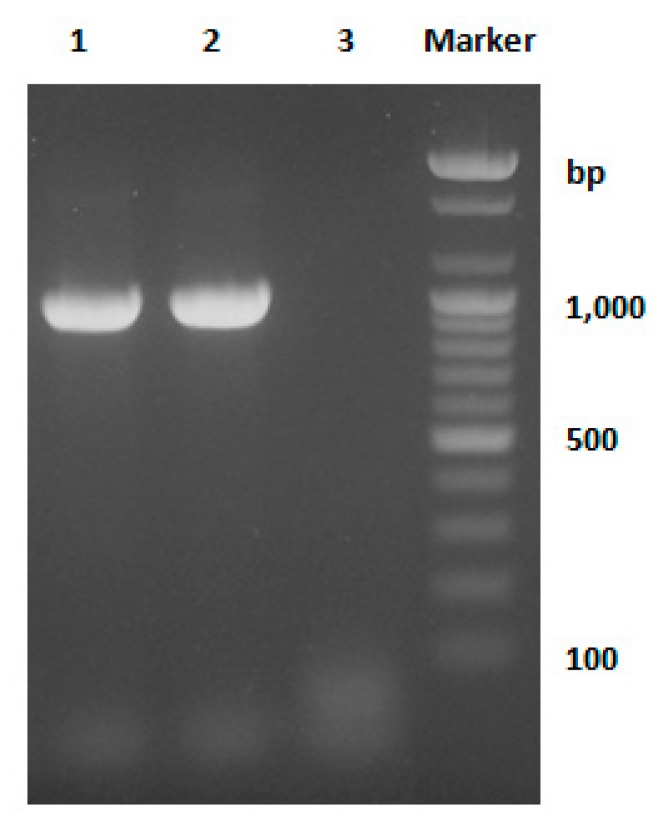
Electrophoretic analysis of PCR products obtained from *Mycoplasma* species. Lane 1: positive control group (*Mycoplasma hyopneumoniae*, KML Mh1 strain); Lane 2: test sample (*Mycoplasma hyopneumoniae*, J101 strain) and Lane 3: negative control group (sterile distilled water).

PCR was performed with primers specific for *Mycoplasma hyopneumoniae* p36. As a result, 948 bp PCR band for the p36 gene was identified in the experimental group (Lane 2) as in the positive control group (Lane 1). Therefore, it was confirmed that the sample used in the experiment was *Mycoplasma hyopneumoniae* (Figure 2).

### 3.3. Pseudo-Ternary Phase Diagram and Self-Emulsification Study

**Figure 3:**
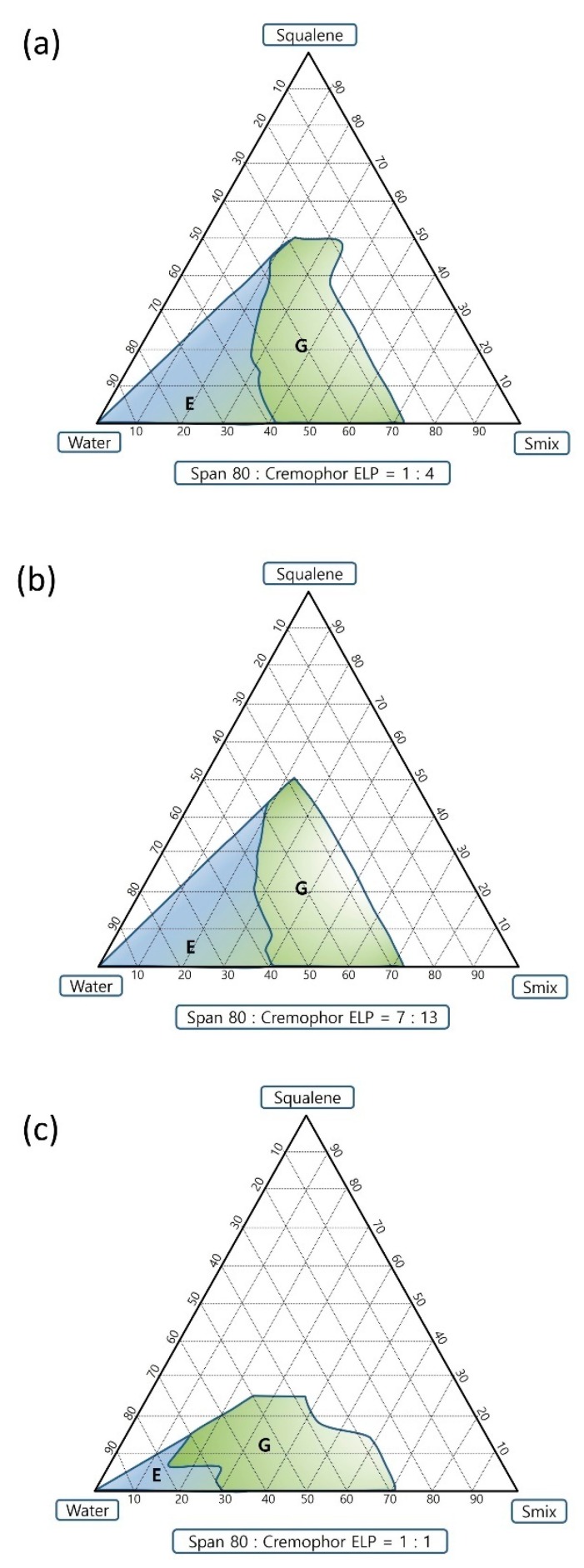
Pseudo-ternary phase diagrams showing the efficient self-emulsification region. The ratios of Span 80 to Cremophor ELP are (**a**) 1:4 (**b**) 7:13 and (**c**) 1:1.

Ratios 1:4 and 7:13 of Span 80 to Cremophor ELP exhibited almost similar efficient self-emulsification areas whereas the ratio 1:1 showed a smaller efficient self-emulsification area (Figure 3). The ratio 1:4 showed the largest gel area followed by ratios 7:13 and 1:1. Ratios 1:4, 7:13 and 1:1 have resultant calculated HLB values of 11.26, 9.96 and 8.65, respectively, considering the HLB (hydrophilic–lipophilic balance) value of Span 80 as 4.3 [20] and Cremophor ELP as 13 (ranged from 12 to 14) [21]. The E region of the ratio 7:13 was chosen to prepare emulsion adjuvants. The visual grade was “A” for the self-emulsification study done using deionized water and the visual grade was “B” for the self-emulsification tests done using 0.01 and 0.02% (*w*/*v*) carbomer solutions of C-971P NF and C-940 grades as shown in Table 3. This might be due to three-dimensional polymeric network formed by carbomer in aqueous phase of the o/w emulsion [22].

### 3.4. Preparation and Characterization of Emulsion Adjuvants

The ratio 7:13 was selected and emulsions containing 3%, 5% and 10% of squalene oil were prepared from the emulsion (E) region with deionized water and 0.01 and 0.02% (*w*/*v*) carbomer solutions of C-971P NF and C-940 grades. Carbomers can enhance cellular immunity when used in vaccine formulations [23]. Therefore, carbomer solutions were also used to prepare emulsions. Variation in particle size of squalene oil emulsions over the period of six months are given in Table 4**.** The majority of emulsions had droplet sizes in the submicron size range and maintenance of their sizes were observed for the period of six months, indicating the stability of the emulsions. Some of them exhibited a slight decrease in particle size with time. Change in the zeta potential of squalene oil emulsions over the period of six months are given in Table 4. Most of them had zeta potential values between −40 mV and 0 mV and, upon gentle shaking, emulsions were easily re-dispersible and could maintain their homogeneity, indicating the stability of the emulsions.

### 3.5. Toxicity Evaluation

**Figure 4:**
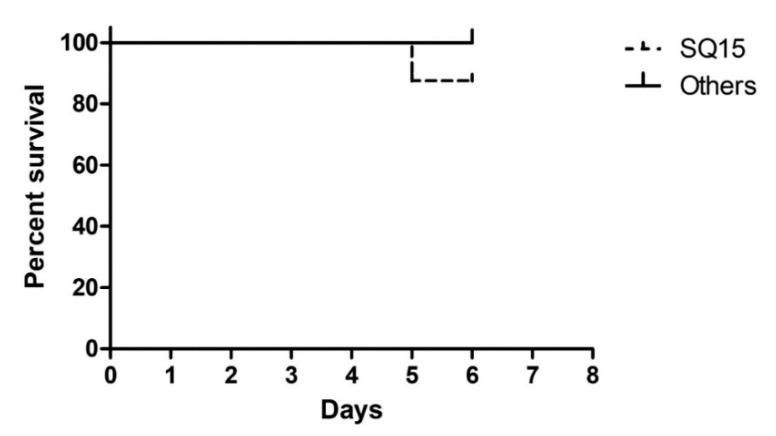
Kaplan–Meier analysis plot showing the toxicity evaluation of emulsions.

Mortality of BALB/c mice was not observed in emulsion adjuvants that did not contain carbomer (SQ1-SQ3) along with other carbomer containing emulsion adjuvants (SQ4-SQ14), throughout the study period. Only the SQ15 group, containing the carbomer (C-940)-based emulsion, caused the death of one mouse (Figure 4).

### 3.6. Determination of Adjuvant-Dependent Antibody Subclasses

**Figure 5:**
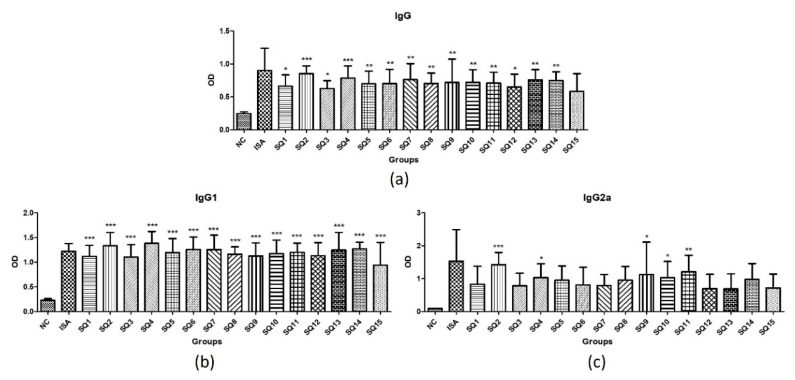
*Mycoplasma hyopneumoniae*-specific antibody responses in immunized mice. (**a**) IgG titer (**b**) IgG1 titer and (**c**) IgG2a titer. Differences were considered statistically significant at * *p* < 0.05, ** *p* < 0.01, and *** *p* < 0.001 vs. NC. Where NC is the negative control that uses only inactivated antigen without adjuvant and it is composed of PCV2 + *Mycoplasma hyopneumoniae* mixed antigens; ISA is the positive control vaccine formulation that contains Montanide ISA^TM^ 201 as adjuvant; and SQ is the test vaccine formulation that contains a squalene oil-based emulsion as adjuvant.

This experiment was conducted not to compare the absolute values of IgG, IgG1 and IgG2a, but to see if they stimulated IgG1 and IgG2a simultaneously. Vaccine adjuvant such as aluminum gels hardly stimulate IgG2a and is specific for humoral immune stimulation because they stimulate only IgG1. On the other hand, oil-based adjuvant such as Montanide ISA^TM^ 201 also stimulate IgG2a, which is known to have a cellular immune stimulating effect. Both SQ1-15 and Montanide ISA^TM^ 201 stimulated IgG, IgG1 and IgG2a at higher levels than NC. As a result, the squalene oil-based candidate vaccine adjuvants of the present research prepared on the basis of the SEDDS were found to have an immune stimulation mechanism similar to that of the currently commercially available Montanide ISA^TM^ 201 (Figure 5).

### 3.7. Measurement of Mycoplasma hyopneumoniae Specific Antibody Level

**Figure 6:**
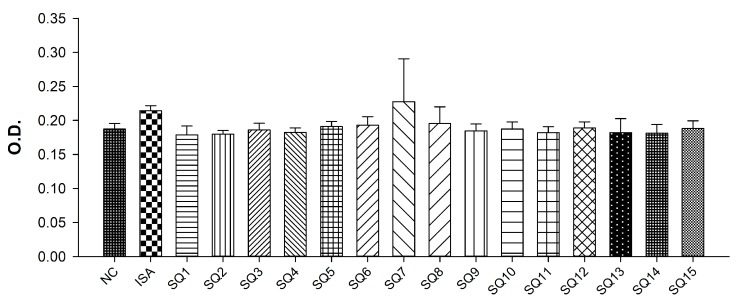
*Mycoplasma* antibody formation results. Because the OD value of SQ 7 is showing an equivalent or more effective performance than ISA, it is expected that SQ7 will show comparable antibody formation like Montanide ISA^TM^ 201. Where NC is the negative control is a serum obtained from non-immunized mice; ISA is the positive control vaccine formulation that contains Montanide ISA^TM^ 201 as adjuvant; and SQ is the test vaccine formulation that contains squalene oil-based emulsion as adjuvant.

The average OD value of the non-inoculated control group (NC) diluted 10-folds was 0.1870, which was slightly lower than the average OD value of 0.1875 of the standard negative sample supplied from Komipharm, and the antibody value of the NC group was confirmed to be 10 times or less. From the result, we found that SQ5-8, 12 and 15 showed a more than 512-fold antibody titer compared to the standard negative sample (Figure 6).

## 4. Discussion

*Mycoplasma hyopneumoniae* expresses several characteristic proteins with high immunogenicity. Among them, p36, a cytoplasmic protein, p46, p65, and p74, cell membrane proteins, and p97, a cell adhesion protein, are typical. P36, the cytoplasmic protein of *Mycoplasma hyopneumoniae*, shows very high concordance in different *Mycoplasma hyopneumoniae* strains scattered around the world. High-immune serum from p36 recombinant protein is not reactive with other porcine mycoplasmas, such as *Mycoplasma flocculare, Mycoplasma hyorhinis* and *Acholeplasma laidlawii*. It has also been demonstrated that there is no cross-reaction with *Mycoplasma* or *Acholeplasma* isolated from humans or other industrial animals, dogs and cats. Therefore, we conducted a genetic test for p36 because p36 protein is an antigen that determines the species specificity of *Mycoplasma hyopneumoniae* [16].

**Figure 7:**
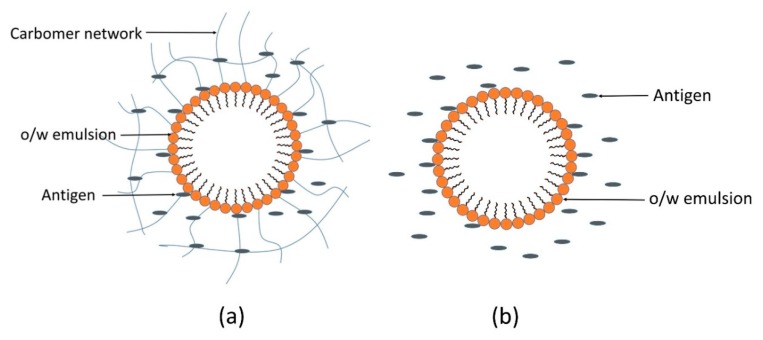
Proposed arrangement of antigens in oil-in-water (o/w) emulsions with and without carbomer. (**a**) Antigens entrapped in a polymer present in the aqueous phase of an o/w emulsion and (**b**) antigens present in the aqueous phase of an o/w emulsion.

The concentration of carbomer administered was 10 times less than that of LD_50_ (median lethal dose). However, the actual dose for the veterinary target animal, pig, is 4-fold higher than that of the mouse, and the pig is 1000-fold larger in size. Thus, all squalene oil-based emulsions were considered as safe and used in the immunization study. Furthermore, in our study, squalene oil, Span 80, Cremophor ELP and two grades of carbomer viz. Carbopol^®^ 971P NF and Carbopol^®^940 were used in low concentrations, and this might be the reason for the safety of emulsion adjuvants. Among different types of immunoglobulins, IgG is one of the most important immunoglobulins, which possesses antibacterial, antivirus, and anti-exotoxin properties. IgG1, IgG2a, IgG2b and IgG3 are different subclasses of IgG. T-lymphocytes and their cytokines regulate the production of IgG subclasses. IFN-γ and IL-2 produced by Th1 lymphocytes improve the production of IgG2a, whereas Th2 cytokines, namely, IL-4 and IL-6, improve the secretion of IgG1 [3]. Although IgG2a values were lower than Montanide ISA^TM^ 201, Figure 5 is showing that squalene oil-based o/w emulsion adjuvants enhanced production of IgG, IgG1 and IgG2a titers compared to the NC group. Thus, it can be suggested that these adjuvants are capable of eliciting both Th1 and Th2 immune responses. In case of o/w emulsions prepared with carbomer solutions, the carbomer forms a three-dimensional polymeric network in the aqueous phase of the o/w emulsion where antigens get entrapped and released from the polymeric network in the aqueous phase of the o/w emulsion, and where antigens get entrapped and released, slowly eliciting immune cells [22]. However, in o/w emulsions prepared with water, antigens are considered to exist simply in the aqueous phase as shown in Figure 7. In this study, emulsion adjuvants prepared with carbomer solutions did not show a significant increase in immunogenic responses in comparison to emulsions prepared with water or the depot effect of carbomer was not proved. It might be due to the short study period conducted in a smaller animal (BALB/c mice) and the use of lower concentrations of carbomer. By observing the characterization parameters and immune responses of emulsion adjuvants and *Mycoplasma hyopneumoniae*-specific antibody formation results, we can suggest SQ7 as a vaccine adjuvant candidate.

## 5. Conclusions

The SEDDS method was successfully used to prepare novel squalene oil-based o/w emulsion adjuvants. Almost all the candidate adjuvants were found to be safe and considered to be used as adjuvants for the *Mycoplasma hyopneumoniae* vaccine. The developed candidate adjuvants prepared on the basis of SEDDS technology showed stimulation of IgG, IgG1 and IgG2a, which reflects they are capable of eliciting both Th1 and Th2-mediated immune responses responsible for IgG2a-based cellular immunity and IgG1-based humoral immunity, respectively. Of which the immune stimulating effect of SQ 7 was considered as comparable with the commercial product—Montanide ISA^TM^ 201. Thus, SEDDS could be a simple and industrially advanced formulation method for the preparation of o/w emulsion adjuvants with an effective immune-boosting effect.

## Figures and Tables

**Table 1:** Composition of squalene oil-based emulsions prepared using self-emulsifying drug delivery system (SEDDS) technology.

Ingredients	SQ1	SQ2	SQ3	SQ4	SQ5	SQ6	SQ7	SQ8	SQ9	SQ10	SQ11	SQ12	SQ13	SQ14	SQ15
Span 80	2.4	4.1	8.2	2.4	4.1	8.2	2.4	4.1	8.2	2.4	4.1	8.2	2.4	4.1	8.2
Cremophor ELP	4.6	7.6	15.1	4.6	7.6	15.1	4.6	7.6	15.1	4.6	7.6	15.1	4.6	7.6	15.1
Squalene oil	3.0	5.0	10.0	3.0	5.0	10.0	3.0	5.0	10.0	3.0	5.0	10.0	3.0	5.0	10.0
Water	90.0	83.3	66.7												
Carbopol^®^C-971P NF sol’n (0.01% *w*/*v*)				90.0	83.3	66.7									
Carbopol^®^971PNF sol’n (0.02% *w*/*v*)							90.0	83.3	66.7						
Carbopol^®^940 sol’n (0.01% *w*/*v*)										90.0	83.3	66.7			
Carbopol^®^940 sol’n (0.02% *w*/*v*)													90.0	83.3	66.7
Total	100.0	100.0	100.0	100.0	100.0	100.0	100.0	100.0	100.0	100.0	100.0	100.0	100.0	100.0	100.0

**Table 2:** Optical and microscopic observation for *Mycoplasma hyopneumoniae* after 14 days.

	Negative Control	*Mycoplasma hyopneumoniae*	Positive Control (*Mycoplasma gallisepticum*)
*Mycoplasma* broth	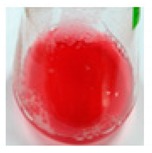	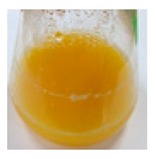	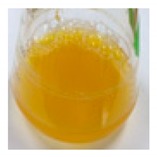
*Mycoplasma* agar	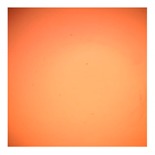	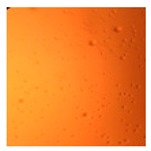	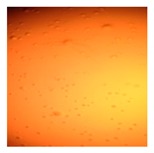

**Table 3:** Results of the self-emulsification study.

Sample Code	Percentage Composition of SEDDS		Formulations Used for Self-Emulsification Test	Visual Grade
	S_mix_	Squalene Oil		
SQ1-SQ3	70%	30%	Water	A
SQ4-SQ6	70%	30%	C-971P NF solution (0.01% *w*/*v*)	B
SQ7-SQ9	70%	30%	C-971P NF solution (0.02% *w*/*v*)	B
SQ10-SQ12	70%	30%	C-940 solution (0.01% *w*/*v*)	B
SQ13-SQ15	70%	30%	C-940 solution (0.02% *w*/*v*)	B

**Table 4:** Change of particle size and zeta potential of squalene oil-based emulsions over the period of six months (mean ± SE).

Sample Code	Particle Size (nm)				Zeta Potential (mV)			
	0 Month	1 Month	3 Months	6 Months	0 Month	1 Month	3 Months	6 Months
SQ1	1589.9 ± 211.3	911.5 ± 148.5	1350.8 ± 74.8	649.6 ± 54.4	−43.58 ± 1.44	0.19 ± 6.39	17.63 ± 13.50	1.47 ± 10.11
SQ2	1211.4 ± 52.0	900.6 ± 56.5	1082.0 ± 22.7	750.9 ± 52.5	−25.24 ± 0.83	−18.23 ± 2.98	−10.47 ± 0.35	−6.41 ± 5.36
SQ3	690.7 ± 36.6	427.5 ± 17.4	557.1 ± 20.8	457.1 ± 19.6	−29.08 ± 3.50	−8.44 ± 5.64	−6.39 ± 8.33	11.11 ± 18.60
SQ4	100.1 ± 15.2	80.7 ± 3.1	228.1 ± 48.2	402.7 ± 50.4	−10.86 ± 1.75	−15.71 ± 2.65	11.85 ± 7.72	−10.81 ± 2.21
SQ5	371.4 ± 179.0	228.3 ± 62.4	455.6 ± 112.3	410.4 ± 65.6	−25.11 ± 0.65	−18.86 ± 1.17	−4.81 ± 7.72	−7.94 ± 1.62
SQ6	1438.4 ± 75.1	570.6 ± 19.3	798.6 ± 18.0	570.9 ± 28.8	−31.86 ± 2.16	−13.22 ± 3.94	−10.29 ± 11.92	−0.61 ± 13.35
SQ7	45.2 ± 0.5	49.6 ± 0.6	70.5 ± 2.1	90.2 ± 3.3	−0.59 ± 8.16	−16.96 ± 1.46	15.82 ± 4.63	6.24 ± 9.56
SQ8	1284.1 ± 31.7	567.2 ± 32.2	625.1 ± 59.4	591.1 ± 60.7	2.84 ± 11.48	−13.84 ± 0.97	−10.79 ± 10.38	−9.18 ± 1.93
SQ9	862.4 ± 35.2	457.5 ± 4.7	398.6 ± 8.6	468.7 ± 34.3	−23.13 ± 5.12	−12.86 ± 1.88	−6.15 ± 4.57	−4.08 ± 11.54
SQ10	2483.4 ± 200.9	1951.5 ± 172.0	1722.7 ± 62.6	700.3 ± 97.5	6.48 ± 8.24	1.21 ± 11.07	1.78 ± 10.73	−11.61 ± 0.50
SQ11	335.8 ± 59.5	765.3 ± 54.1	721.2 ± 64.7	483.9 ± 22.2	−15.98 ± 1.67	3.95 ± 6.57	−4.47 ± 10.36	−9.04 ± 1.29
SQ12	920.4 ± 45.6	622.5 ± 32.2	739.1 ± 37.4	537.4 ± 15.1	−24.44 ± 1.74	−10.79 ± 7.83	−4.05 ± 7.56	7.55 ± 1.76
SQ13	697.4 ± 91.3	831.7 ± 82.6	856.1 ± 38.7	621.4 ± 23.3	11.55 ± 1.59	10.18 ± 4.00	−4.59 ± 12.61	−12.98 ± 2.52
SQ14	217.9 ± 21.9	353.4 ± 70.4	1086.5 ± 45.8	683.0 ± 44.2	−9.18 ± 12.24	0.59 ± 13.56	1.89 ± 18.59	−11.31 ± 3.22
SQ15	1211.4 ± 90.8	968.0 ± 10.8	899.5 ± 54.4	582.1 ± 28.5	−34.17 ± 2.56	−14.65 ± 4.33	2.14 ± 9.13	−7.56 ± 10.96
